# Transcriptomic Alterations of Canine Histiocytic Sarcoma Cells in Response to Different Stressors

**DOI:** 10.3390/ijms26146629

**Published:** 2025-07-10

**Authors:** Thanaporn Asawapattanakul, Klaus Schughart, Maren von Köckritz-Blickwede, Federico Armando, Peter Claus, Wolfgang Baumgärtner, Christina Puff

**Affiliations:** 1 Department of Pathology, University of Veterinary Medicine Hannover, 30559 Hannover, Germany; thanaporn.asawapattanakul@tiho-hannover.de (T.A.); federico.armando@unipr.it (F.A.); christina.puff@tiho-hannover.de (C.P.); 2 Center for Systems Neuroscience (ZSN), 30559 Hannover, Germany; claus.peter@mh-hannover.de; 3 Institute of Virology Münster, University of Münster, 48149 Münster, Germany; labschughart@online.de; 4 Department of Microbiology, Immunology and Biochemistry, University of Tennessee Health Science Center, Memphis, TN 38163, USA; 5 Institute of Biochemistry, University of Veterinary Medicine Hannover, 30559 Hannover, Germany; maren.von.koeckritz-blickwede@tiho-hannover.de; 6 Research Center for Emerging Infections and Zoonoses (RIZ), 30559 Hannover, Germany; 7 Unit of Pathology, Department of Veterinary Science, University of Parma, 43126 Parma, Italy; 8 Laboratory of Molecular Neurosciences, Department of Psychiatry, Social Psychiatry and Psychotherapy, Hannover Medical School, 30625 Hannover, Germany

**Keywords:** hypoxia, metabolism, RNA sequencing, starvation, stress, tumor zones

## Abstract

Canine histiocytic sarcoma (HS) is a rare tumor with a poor prognosis. Rapid tumor growth often causes central hypoxia and starvation, impacting tumor progression. In the present study, HS cells were cultured under hypoxia and starvation for 1 and 3 days, simulating intermediate and central tumor zones, respectively. Cells were counted at each time point, followed by RNAseq analysis. Only hypoxia significantly reduced the cell number (*p* < 0.05). Short-term hypoxia altered 1645 differentially expressed genes (DEGs). Upregulated genes belonged to vasculature development, and downregulated genes to cell cycle processes. Short-term starvation affected 157 genes, mainly involving responses to stimuli. Prolonged hypoxia and starvation induced 1301 and 836 DEGs, respectively. Prolonged hypoxia upregulated genes mainly involved in immune responses, response to stimulus, adhesion, and angiogenesis. Prolonged starvation upregulated genes associated with signaling, adhesion, circulatory system development, and response to stimulus. Lipid metabolism and cell cycle pathways were downregulated under prolonged hypoxia and starvation, respectively. KEGG “pathways in cancer” were enriched under all conditions (adjusted *p*-values < 0.05). These findings indicate that hypoxia and starvation significantly alter the expression of genes involved in tumor progression. Further studies, namely post-translational analyses, are needed to elucidate the functional impact of these changes and identify potential therapeutic targets.

## 1. Introduction

Canine histiocytic sarcoma (HS) is a malignant solid tumor in dogs, which originates from the neoplastic proliferation of macrophages or dendritic cells. HS can occur as a single mass (localized HS) or be spread throughout several organs (disseminated HS), such as the spleen, liver, lungs, lymph nodes, bone marrow, and central nervous system [1].

HS affecting the central nervous system (CNS) in dogs may present as a primary or disseminated tumor. Although primary HS in the CNS is uncommon, the prognosis is poor [2], and the range of survival time varies from a few days to several months [3]. The most commonly affected breeds are Rottweilers, Bernese Mountain Dogs, and retrievers. However, Pembroke Welsh Corgis and Shetland Sheepdogs are predisposed to primary CNS HS [2,4].

Tumor hypoxia and starvation are hallmark drivers of malignancy that have been observed in various solid tumors, particularly in central tumor areas, considered to be a potential therapeutic challenge [5,6,7]. These stressors promote tumor progression by modulating the tumor microenvironment (TME) by activating specific receptors associated with many biological processes, including several cancer hallmarks, such as proliferation, angiogenesis, tissue invasion and metastasis, genomic instability, metabolism, and immune evasion [8,9,10,11].

Intratumoral hypoxia can result from an imbalance between oxygen availability and consumption, mainly due to abnormal microcirculation and poor diffusion conditions. Tissue with an oxygen partial pressure (pO_2_) below 10 mmHg, compared to 40–60 mmHg in most normal tissues, is referred to as hypoxic tissue [12,13]. For the analysis of intratumoral hypoxia, many methods are used in vivo. Invasive methods to identify and quantify the pO_2_ levels include Eppendorf needle electrode measurements [7,14,15] and immunohistochemistry, which is used as an indirect method to visualize and quantify the expression of proteins related to oxygen. Furthermore, non-invasive methods applying positron emission tomography (PET) [7,16], single-photon emission computed tomography (SPECT) [17], and magnetic resonance imaging (MRI) [18,19,20,21,22,23] can additionally be used in vivo. Hypoxia activates the hypoxia inducible factor (HIF), which is a key regulator of cellular adaptation to this stressor in the TME. This transcription factor plays an important role by changing the expression of genes involved in angiogenesis, energy metabolism, oxygen consumption, invasion, and metastasis [24,25,26]. Additionally, a reduced mitochondrial oxygen consumption and ATP production in hypoxic and starving neoplastic cells can impair active transport processes, leading to several effects, including collapse of sodium and potassium gradients, membrane depolarization, increased chloride uptake, cell swelling, elevated cytosolic calcium levels, and decreased cytosolic pH, causing intracellular acidosis [27]. These alterations not only affect cell functions but also impact the TME, leading to more aggressive tumor behavior [28,29,30,31].

Hypoxia and starvation, both well-recognized cellular stressors, are often present inside rapidly growing tumors due to inadequate blood supply and high metabolic demand [32,33]. Starving cells can be identified via several methods, including transmission electron microscopy, which reveals an accumulation of cytoplasmic lipid droplets (fatty change), mitochondrial function assays, and detection of starvation-induced autophagy, such as LC3 protein expression, via immunohistochemistry or immunofluorescence [34,35]. This starvation can trigger cellular responses including autophagy, apoptosis, and metabolic adaptations [33,36,37]. Autophagy, which allows cells to recycle and generate energy, is crucial for tumor growth [38]. Without autophagy, these cells cannot adapt to stress induced by nutrient deprivation, leading to increased apoptosis or necrosis [36,39]. Therefore, the TME plays a crucial role in tumor development. In solid tumors like HS, rapid cell proliferation often overcomes vascular supply, leading to regions of hypoxia and nutrient deprivation, especially in central tumor zones. These conditions activate adaptive stress responses in tumor cells, driving changes in gene expression. Although hypoxia- and starvation-induced effects have become one of the most important research areas in recent years to improve the understanding of cellular responses and to develop potential therapeutic strategies against neoplasms [10,13,36,40,41,42], few studies have deciphered the differences in zones that possess varying levels of oxygen and nutrients within solid tumors. The differences in these compartments could lead to variations in cellular responses that could influence the effectiveness of treatment schemes [43,44]. Furthermore, many studies have demonstrated a high conservation of transcriptional profiles across species, enabling the identification of shared oncogenic pathways and potential targets relevant to both canine and human disease [45,46,47]. This includes the impacts on the tumor microenvironment, like the consequences of hypoxia on microRNA expression in canine glioma, which shares significant similarities with human neoplasms [48]. In the field of tumor biology, spontaneous neoplasms in companion animals are recognized as appropriate models for the investigation of human tumors, especially in rare neoplasms where it is difficult to gain deeper mechanistic insights and develop therapeutic options [46,47,49,50]. Nevertheless, the molecular features of canine histiocytic sarcoma, as determined by gene expression analysis, indicate an activation of the ERK and Akt [51] as well as the NF-κB pathways [52,53].

Consequently, this study aimed to investigate stress-induced transcriptomic dysregulations in canine histiocytic sarcoma cells, simulating various areas within a solid tumor. This research focused on identifying key gene expression changes that occur under hypoxic and starving conditions of various durations.

## 2. Results

### 2.1. Proliferation Assay

**Figure 1:**
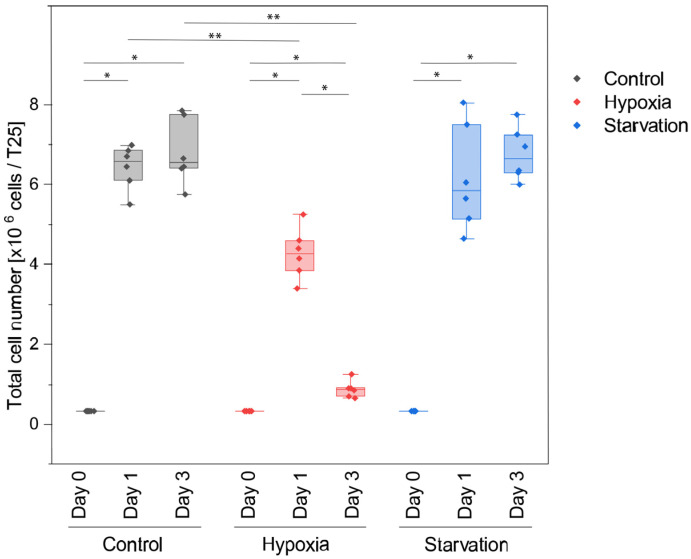
Proliferation assay of DH82 cells cultured under control, hypoxic, and starving conditions. Depicted are minimum, 25% quartile, median, 75% quartile, and maximum. * *p* < 0.05, ** *p* < 0.01.

To evaluate the proliferation of canine histiocytic sarcoma cells (DH82 cells), the cell number was determined at day 1 and day 3 under the respective culture conditions, and compared to the number of initially seeded cells per flask (0.33 × 10^6^ cells/T25) (Table 1, Figure 1).

Overall, statistical analysis revealed a change in cell counts after culturing for 3 days (*p* < 0.01). When comparing the pairs, statistically significant changes were observed from day 0 to day 1, and from day 0 to day 3. The number of cells significantly increased over time, independent of the condition (*p* < 0.05). From day 1 to day 3, a marked decrease in the total cell number was observed under hypoxia (*p* = 0.028), while no statistically significant differences were found under starving and control conditions (*p* > 0.05). Moreover, significant differences in proliferation rates were noted in hypoxic groups compared to control groups on both, day 1 and day 3 (*p* = 0.004 for both comparisons). In contrast, under starvation, no statistically significant differences were found compared to controls.

### 2.2. Transcriptomic Changes in Canine Histiocytic Sarcoma Cells

**Figure 2:**
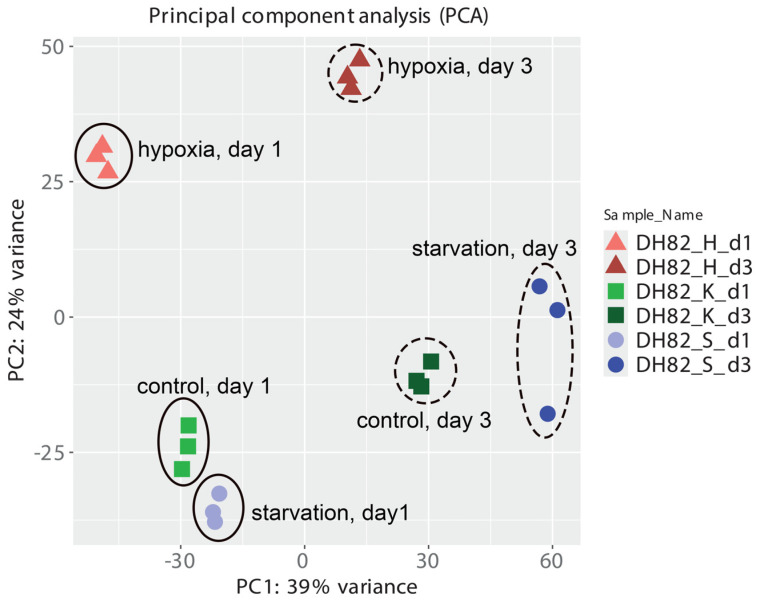
Principal component analysis plot of transcriptomic data, showing that all three replicates of all six groups clustered separately. DH82: canine histiocytic sarcoma cells, K: control, H: hypoxia, S: starvation, d1: day 1, d3: day 3.

To reveal the transcriptomic expression between canine histiocytic sarcoma cells (DH82) cultured under different conditions, the transcriptional level of the 11,373 genes was compared. A principal component (PC) analysis of DH82 cells cultured under all conditions was performed using normalized data. It indicated expected grouping among replicates with samples and sample groups spread across two PCs (PC1 and PC2). Of the total variation, 39% was explained by the first principal component (PC1), and PC2 accounted for 24% of the variation. Figure 2 displays the PC analysis plot of samples, showing clear clustering of the three replicates and separation across all six groups.

### 2.3. Identification of Differentially Expressed Genes (DEGs)

At the transcriptional level, a total of 11,373 genes was detected and annotated by aligning the sequencing reads to the canine reference genome. Transcripts were then tested for differential expression; genes showing an absolute log_2_ fold change > 1 and an adjusted *p*-value < 0.05 were selected as DEGs for this study (Table 2).

#### 2.3.1. Hypoxia

**Figure 3:**
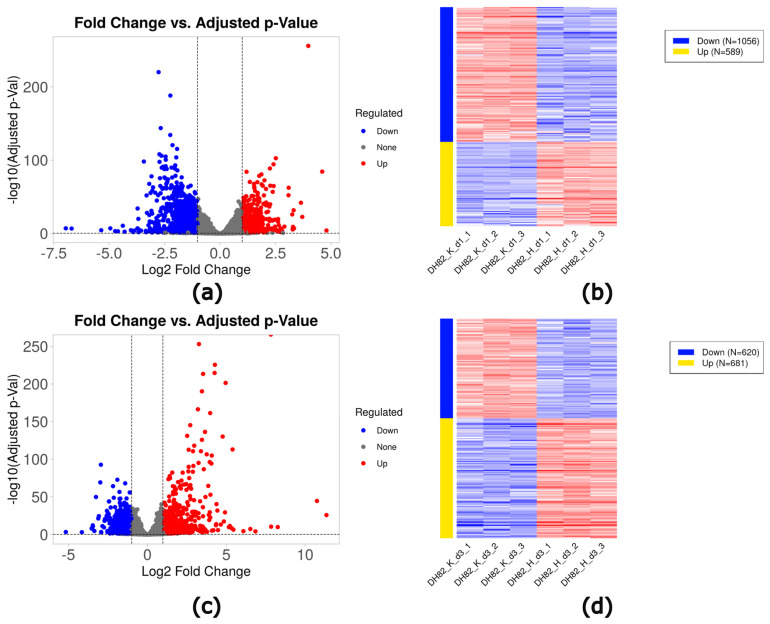
Volcano plots (**a**,**c**) and heat maps (**b**,**d**) of DEGs in DH82 cells cultured under hypoxia for a short time frame (1d; (**a**,**b**)) and after prolonged exposure (3d; (**c**,**d**)) compared to control conditions. Volcano plots. Significantly up- and downregulated genes (adjusted *p*-value ≤ 0.05; log_10_ adjusted *p*-value ≤ 1.3) are indicated by red (log_2_FC > 1) and blue (log_2_FC < −1), respectively. DH82: canine histiocytic sarcoma cells, K: control, H: hypoxia, S: starvation, d1: day 1, d3: day 3.

In short-term (1d) hypoxic cultures, 1645 DEGs (589 upregulated and 1056 downregulated) were identified compared to controls (Table 2, Figure 3a,b). Prolonged exposure (3d) to hypoxia led to 1301 DEGs (681 upregulated and 620 downregulated) compared to controls (Table 2, Figure 3c,d). Top ten genes were shown in Tables S1 and S2.

**Figure 4:**
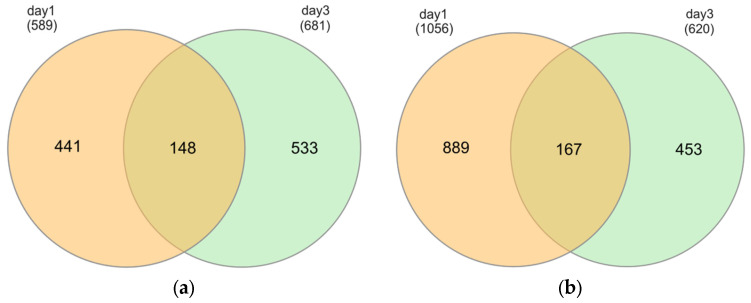
Venn diagram of common DEGs in DH82 cells cultured under hypoxic conditions for a short time frame (1d) and after prolonged exposure (3d). (**a**) Upregulated common DEGs. (**b**) Downregulated common DEGs.

There was an overlap of DEGs between short-term and prolonged exposure to hypoxia compared to the corresponding controls. In total, 148 transcripts (25% of upregulated DEGs for short-term hypoxia) were also upregulated under prolonged exposure to hypoxia, while 167 common genes (15.8% of downregulated DEGs for short-term hypoxia) were also downregulated at the later time point (Figure 4a,b).

#### 2.3.2. Starvation

**Figure 5:**
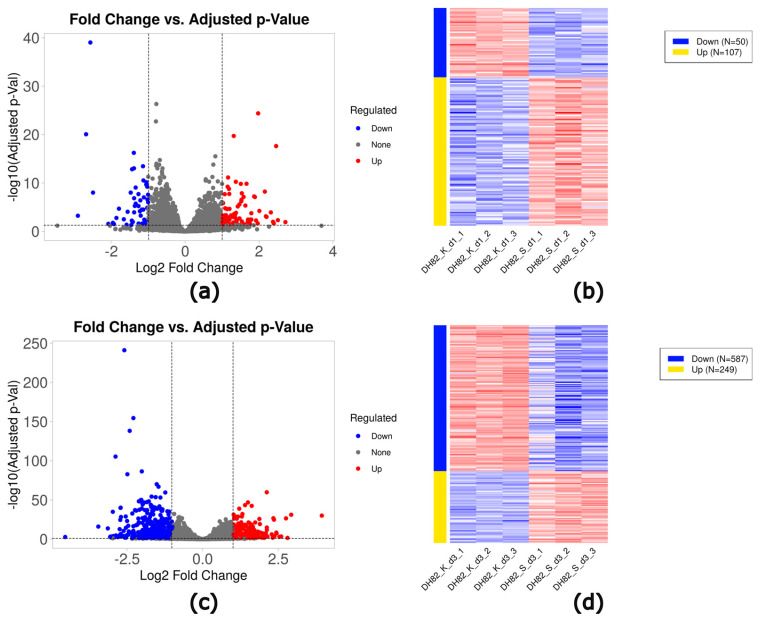
Volcano plots (**a**,**c**) and heat maps (**b**,**d**) of DEGs in DH82 cells cultured under starvation for a short time frame (1d; (**a**,**b**)) and prolonged exposure (3d; (**c**,**d**)) compared to control conditions. Volcano plots. Significantly up- and downregulated genes (adjusted *p*-value ≤ 0.05; log_10_ adjusted *p*-value ≤ 1.3) are indicated by red (log_2_FC > 1) and blue (log_2_FC < −1), respectively. DH82: canine histiocytic sarcoma cells, K: control, H: hypoxia, S: starvation, d1: day 1, d3: day 3.

In short-term (1d) starvation, 157 DEGs (107 upregulated and 50 downregulated) were identified compared to controls (Table 2, Figure 5a,b). In prolonged starvation (3d), 836 DEGs (249 upregulated and 587 downregulated) were found compared to controls (Table 2, Figure 5c,d). Top ten genes were shown in Tables S1 and S2.

**Figure 6:**
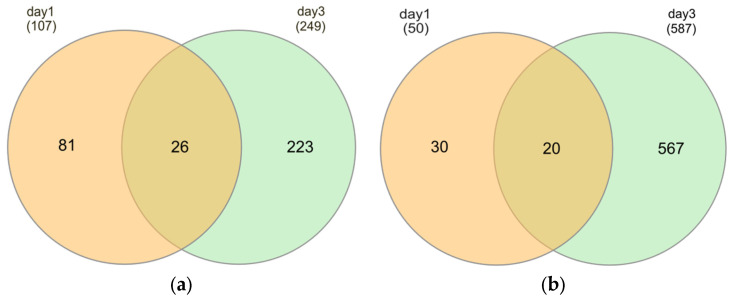
Venn diagram of common DEGs in DH82 cells cultured under starvation conditions for short time frames (1d) and after prolonged exposure (3d). (**a**) Upregulated common DEGs. (**b**) Downregulated common DEGs.

However, following starvation, there was a significant overlap of differentially expressed genes (DEGs) between short-term and prolonged exposure groups. A total of 26 (24.3%) upregulated and 20 (40%) downregulated DEGs in the short-term starvation group were also regulated in the prolonged starvation group (Figure 6a,b).

#### 2.3.3. Time Effects

**Figure 7:**
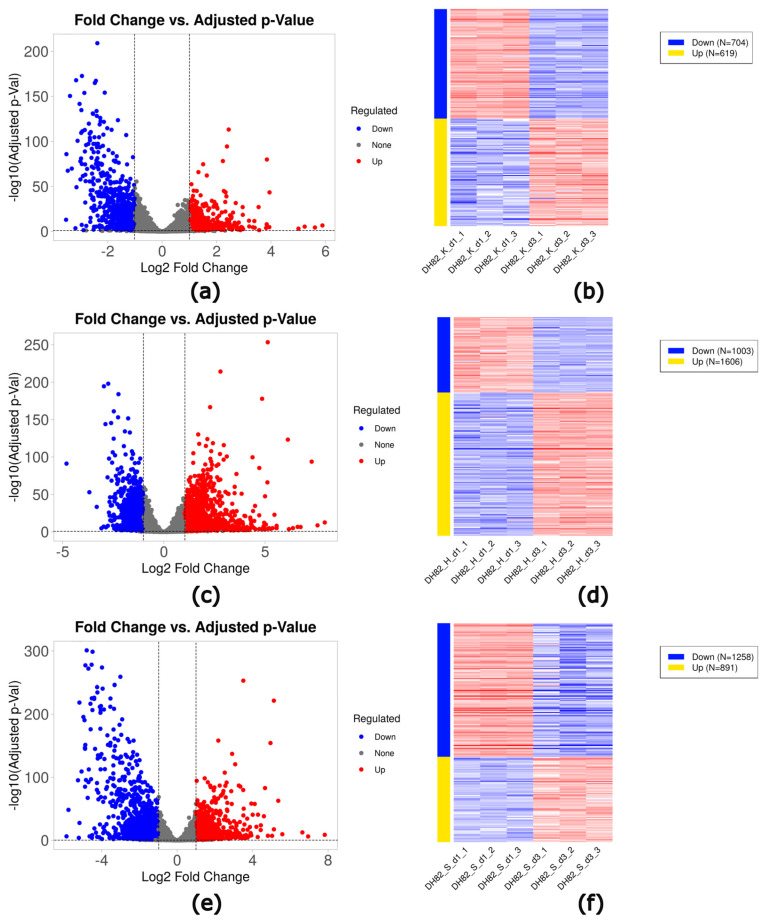
Volcano plots (**a**,**c**,**e**) and heat maps (**b**,**d**,**f**) of DEGs in DH82 cells cultured under control (**a**,**b**), hypoxia (**c**,**d**), and starvation (**e**,**f**) after prolonged exposure (3d) compared to a short time exposure (1d). Volcano plots. Significantly up- and downregulated genes (adjusted *p*-value ≤ 0.05; log_10_ adjusted *p*-value ≤ 1.3) are indicated by red (log_2_FC > 1) and blue (log_2_FC < −1), respectively. DH82: canine histiocytic sarcoma cells, K: control, H: hypoxia, S: starvation, d1: day 1, d3: day 3.

A high number of DEGs was observed in cultures exposed to prolonged hypoxia and starvation. Under control conditions, 1323 DEGs, with 619 up- and 704 downregulated genes, were observed at day 3 compared to day 1. Under hypoxic conditions, 2609 DEGs (1606 up- and 1003 downregulated genes) were found, comparing the expression at day 3 with day 1. Likewise, starving cultures exhibited 2149 DEGs (891 up- and 1258 downregulated genes), comparing the expression at day 3 with day 1 (Table 2, Figure 7).

### 2.4. Functional Enrichment Analysis

#### 2.4.1. Hypoxia

In short-term hypoxia (1d), an enrichment analysis for gene ontology (GO) biological process terms revealed 729 significantly up- and 211 downregulated terms, respectively, compared to controls (Table S3). Interestingly, GO biological processes associated with circulatory system development, regulation of communication, regulation of signal transduction, and cell adhesion were upregulated in DH82 cells cultured under hypoxia for 1d (short-term) and 3d (prolonged exposure) compared to the corresponding controls (Table 3). Furthermore, altered GO terms of upregulated DEGs in prolonged hypoxia were also associated with the host response, including immune response, response to stimulus, and inflammatory response. On the other hand, GO terms associated with cell cycle, cell division, and genome instability (DNA repair GO term) were downregulated in short-term hypoxia. The most striking observation emerging from long-term hypoxia was downregulation of terms related to lipid metabolic processes (Table 3). In addition, cell activation, response to stimulus, and cell communication were also observed in altered GO terms of downregulated DEGs in prolonged exposure to hypoxia. Detailed information about the GO terms is presented in Table S4.

KEGG (Kyoto Encyclopedia of Genes and Genomes) pathway enrichment analysis identified a total of 72 enriched KEGG pathways in cells exposed to short-term hypoxia, of which 67 were upregulated and 5 downregulated. A total of 52 KEGG pathways were enriched in prolonged exposure to hypoxia, of which 51 were upregulated and 1 downregulated (Table S3). The pathways for each group are shown in Table 4. Regarding the associations with KEGG pathways, “pathways in cancer” was one of the most overrepresented terms and is summarized in Figure S2 for short-term and Figure S3 for prolonged exposure to hypoxia.

Furthermore, KEGG pathways of upregulated DEGs in short-term hypoxia identified a dysregulation in focal adhesion, endocytosis, and some signaling pathways, including AGE-RAGE, HIF-1, TNF, MAPK, NF-κB, and PI3K-Akt. In the case of prolonged hypoxia, KEGG pathways including cytokine–cytokine receptor interaction, cell adhesion molecules, and signaling pathways, like IL-17, TNF, MAPK, NF-κB, JAK-STAT, and NOD-like receptor signaling, were also found to be upregulated. Conversely, the pathways of cell cycle, DNA replication, homologous recombination, and mismatch repair were downregulated during short-term exposure, while the metabolic pathway was affected during prolonged exposure to hypoxia. Detailed information about regulated KEGG pathways is presented in Table S5.

#### 2.4.2. Starvation

Transcriptomic alterations observed during short-term starvation were related to a few GO terms and pathways. Dysregulation of all DEGs under short-term starvation (1d) mainly involved GO terms associated with cellular responses to stimuli and signaling pathways (Table 3 and Table S4). Interestingly, prolonged exposure to starvation (3d) revealed significant associations with 211 GO terms, including 8 GO terms for upregulated and 203 GO terms for downregulated processes. Importantly, the upregulated GO terms identified were associated with tumorigenesis, like circulatory system development, cell/biological adhesion, the ERK1 and ERK2 cascade, and regulation of the ERK1 and ERK2 cascade, some signaling pathways like the cell surface receptor signaling pathway, as well as cellular response-associated processes to stimuli. In contrast, the downregulated GO terms predominantly encompassed processes linked to the cell cycle process and cell division (Table 3 and Table S4).

The few significant KEGG pathways involved in short-term (two pathways) and prolonged exposure to starvation (three pathways) are shown in Table S5. The two altered KEGG pathways in short-term exposure were associated with downregulated gene expressions linked to complement and coagulation cascades and pathways in cancer (Figure S4). Similarly, pathways in cancer were also downregulated in prolonged exposure to starvation (3d) (Figure S5), along with the cell cycle pathway. However, the complement and coagulation cascades pathways were upregulated in the prolonged exposure group, in contrast to downregulation in short-term exposure. The KEGG pathways in cancer are shown in Figure S4 for short-term and Figure S5 for prolonged exposure to starvation, and detailed information about the pathways is presented in Table S5.

To summarize, limited alterations were present after short-term starvation, while a more extensive regulation was observed after a prolonged exposure (3d) to starvation.

#### 2.4.3. Time Effect

GO analysis revealed that 411, 856, and 552 GO terms were involved in control, hypoxic, and starving cells, respectively, when cultured for 3d compared to short-term cultivation (1d).

In cells cultured under control conditions, the upregulated genes were not associated with significantly enriched GO terms. However, cell cycle processes and related pathways were enriched in the downregulated genes (Table 3 and Table S4).

Transcriptomic alterations in cells exposed to prolonged hypoxia (3d) were related to the overexpression of regulation of gene expression, biosynthetic processes, response to stress, regulation of metabolic processes, and apoptotic processes compared to cells cultured under this condition for 1d (short-term). On the other hand, pathways associated with cellular activation (cell migration, endocytosis, cell activation, phagocytosis), cell adhesion, the immune system, new vessel formation, and response to stimulus were downregulated after 3d compared to 1d (Table 3 and Table S4).

In starved cells, the GO term lipid metabolic process was the top finding for overexpression in DH82 cells after prolonged exposure (3d) compared to short-term (1d) cultures. Additional terms in the subsequent ranking can be found in Table 3. Furthermore, GO terms associated with cell cycle processes, DNA repair, and cell migration were downregulated in cells exposed to prolonged starvation (3d) compared to short-term exposure (1d; Table 3 and Table S4).

Over time, cells displayed a significant downregulation of genes clustering in the steroid biosynthesis KEGG pathway, independent of the culture condition. While no KEGG pathways were enriched in the upregulated genes of cells cultured under control and starving conditions, the downregulated genes were enriched in pathways related to cell cycle, DNA replication, pyrimidine metabolism, mismatch repair, and base excision repair in both control and starving conditions over time. Additionally, other pathways, including homologous recombination, transcriptional misregulation in cancer, and p53 and IL-17 signaling pathways, were observed in control conditions, and ECM–receptor interaction, cytokine–cytokine receptor interaction, the PI3K-Akt signaling pathway, and motor proteins were found in starvation. For cells cultured under hypoxia, notably, genes associated to KEGG pathways involved in cytokine–cytokine receptor interaction and signaling pathways, including JAK-STAT, neurotrophin, and TNF signaling pathways, were upregulated, and KEGG pathways related to cell activation, such as focal adhesion and phagosome, metabolism pathways, glutathione metabolism, ECM–receptor interaction, proteoglycans in cancer, and some signaling pathways, including Rap1 and PI3K-Akt signaling pathways, were downregulated. More detailed information about regulated KEGG pathways is shown in Table 4 and Table S5.

#### 2.4.4. Identification of Common DEGs

**Figure 8:**
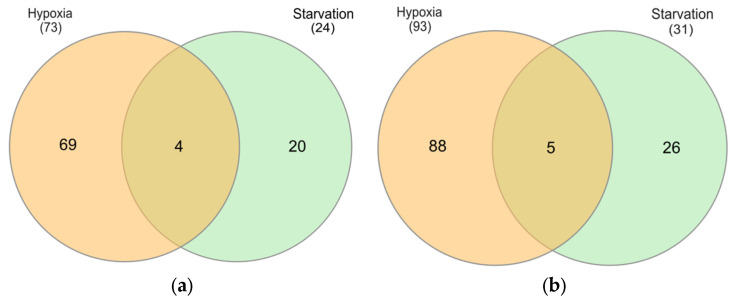
Venn diagram of common upregulated DEGs of hypoxia and starvation in terms of (**a**) circulatory system development and (**b**) cell adhesion.

**Figure 9:**
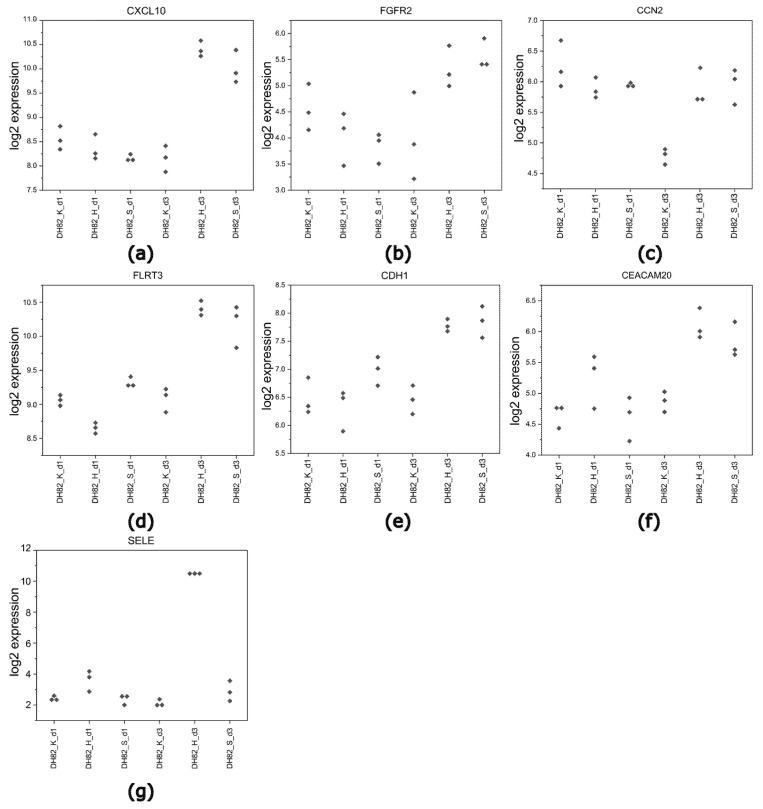
Dot plots representing upregulated genes in the GO terms circulatory system development (**a**–**d**) and cell adhesion (**c**–**g**) in DH82 cells exposed to prolonged hypoxia and starvation. DH82: canine histiocytic sarcoma cells, K: control, H: hypoxia, S: starvation, d1: day 1, d3: day 3.

To identify DEGs, a comparative analysis was performed between hypoxic and starving conditions. At day 3, both conditions showed significant alterations in the upregulation of genes linked to new vessel formation, particularly in the terms circulatory system development and cell adhesion. Identified genes contained 73 upregulated DEGs under hypoxia and 24 DEGs under starvation. Additionally, for the cell adhesion GO term, there were 93 upregulated DEGs in hypoxia and 31 DEGs in starvation. Subsequent analysis indicated that several of these DEGs were shared between the two conditions. The study identified four common DEGs in the circulatory system development in both conditions (*CCN2*, *FLRT3*, *CXCL10*, *FGFR2*), and five common DEGs associated with the cell adhesion term (*SELE*, *CDH1*, *CCN2*, *FLRT3*, *CEACAM20*), as shown in Figure 8. The expression levels per group of the common upregulated DEGs in the circulatory system development and cell adhesion terms are shown in Figure 9.

## 3. Discussion

The present study focuses on transcriptional changes in canine histiocytic sarcoma cells following different stressors, typically observed in the center of solid tumors [54,55,56,57]. These stressors include hypoxia and nutrient deprivation for different time frames, to simulate the conditions in various areas of a solid neoplasm. Tumor cells exposed to stressors for a prolonged time (3 days) represent the central zone of the neoplasm, while a short-term exposure (1d) mirrors the intermediate zone. Cells cultured under standard conditions were used to analyze the optimally supplied peripheral regions of a tumor.

In the present study, cellular stress induced by starvation had no significant effects on cell growth compared to controls, independent of the time point. However, cellular stress induced by starvation can have varying effects on cell growth. While some tumor cells exhibit remarkable tolerance to nutrient deprivation [58], others are significantly affected, leading to cell death [59,60,61]. Izuishi et al. (2000) demonstrated varying levels of tolerance to starvation depending on the cell type [58]. Normal fibroblasts died within 24 h, whereas more than 50% of pancreatic cancer cell lines survived after 48 h, and more than 50% of cells from colon cancer cell lines survived after 36 h [58]. Furthermore, the starvation stress suppresses the cell proliferation of human breast cancer cells [59]. Similarly, in 4T1 breast cancer cells, short-term starvation resulted in increased caspase-3 cleavage and apoptosis [61].

In contrast, a significantly lower number of cells was present in hypoxic DH82 cell cultures, independent of the length of exposure, compared to controls. This points toward a negative impact of hypoxia on cell proliferation and survival in central and intermediate areas of a solid tumor. Furthermore, the analysis revealed that the number of cells was lower in the central zone (day 3). These findings broadly support the work of other studies in this area linking tumor cell proliferation and exposure to hypoxia for short-term [62] and prolonged time frames [63,64]. However, this effect was not sustained with prolonged exposure. Previous research indicates that long-term exposure to hypoxia (14–28 days) increased the proliferative abilities of colon cancer cells (SW480 and HCT116) compared to normoxic conditions, particularly after two weeks of hypoxia [65], as the cells began to adapt, leading to increased survival rates and transcriptomic reprogramming that enabled them to overcome hypoxic conditions [43,65,66].

The present study focuses on transcriptomic alterations induced by hypoxia and starvation, leading to the observed effects in growth behavior. According to the hallmarks of cancer [8,67,68], this study suggests that transcriptomic regulations in canine histiocytic sarcoma cells cultured under hypoxic conditions for a short time frame exhibit an upregulation of genes associated with inducing angiogenesis and metastasis. This indicates that hypoxia triggers various molecular pathways that enhance tumor progression and metastasis [28,29,31,41]. The tumor cells exhibit an adaptation to this hypoxia by trying to increase the number of blood vessels to meet their demand to survive. This finding is consistent with previous results obtained by culturing neoplastic cells under this condition [25,69,70].

Hypoxia inducible factors (HIFs) are a key regulator of the cellular response to hypoxia in the TME. HIF activation promotes angiogenesis [25,70], erythropoiesis, metabolism, and cell survival and proliferation. In some tumor types, hypoxia-induced HIF activation can lead to increased cell survival and proliferation [71,72], potentially counteracting the inhibitory effects of hypoxia on cell proliferation.

The process of the metastasis of tumor cells is complex and involves multiple steps, such as cell detachment, migration, and even colonization. Cell adhesion plays a crucial role in metastasis, influencing these processes. Particularly, cell adhesion molecules (CAMs) are critical for maintaining tissue architecture and enabling cell migration [73,74,75]. This process involves alterations in cell–cell and cell–extracellular matrix (ECM) interactions [76]. The epithelial-to-mesenchymal transition (EMT), a process that enhances cancer cell invasiveness, is marked by changes in the expression of CAMs. For instance, the upregulation of N-cadherin and downregulation of E-cadherin are associated with increased metastatic potential [77,78]. Thus, the role of each gene in enhancing or suppressing metastasis depends on its regulatory state. Moreover, altered expression of CAMs is associated with increased metastatic potential and poor prognosis in various types of tumors such as colon, lung, breast, and prostate cancer [79,80,81]. Furthermore, migration and its regulation are also related to metastasis. A previous study found that the migration of canine histiocytic sarcoma cells is enhanced by hypoxia through the activation of signaling pathways such as ERK and Akt [51]. This is consistent with the findings of the present study, which revealed an upregulation of the PI3K-Akt signaling pathway in hypoxia. The activation of the PI3K-Akt pathway promotes the invasion of various types of neoplastic cells [82,83,84,85]. Moreover, PI3K, AKT, and mTOR proteins also participate in the transcription and translation of HIFs [86].

On the other hand, a decreased expression of genes involved in cell cycle processes, cell division, and genome stability, such as homologous recombination and DNA repair, was noted in the present study. This represents a potential mechanism by which hypoxia influences cell division [87] and promotes genomic instability, for example, because cells have lost the ability to effectively repair DNA damage [43,88,89]. Cowman et al. (2021) reported the downregulation of pathways associated with DNA repair gene expression, including homologous recombination repair, non-homologous end-joining, and mismatch repair, in glioblastoma cell lines under hypoxia [89].

Similarly, in addition to enhancing angiogenesis and metastasis, cells cultured under prolonged hypoxia (3d) also exhibited an increased expression of genes associated with the regulation of the immune response and inflammatory response, along with a decreased expression in genes associated with metabolism, particularly lipid metabolic processes.

Tumor-promoting inflammation is another feature often associated with the malignant behavior of neoplastic cells, which can create a microenvironment favoring tumor progression and resistance to therapy [8]. The present study revealed an upregulation of the inflammatory response GO term and inflammatory response-associated KEGG pathways in prolonged exposure to hypoxia, including tumor necrosis factor (TNF) and nuclear factor kappa B (NF-κB) signaling pathways, which play a crucial role in mediating inflammation and immune responses, suggesting that hypoxic conditions may further exacerbate tumorigenesis through these mechanisms [90]. Furthermore, the activation of NF-κB leads to tumor cell invasion and metastasis [30]. This is promoted by the expression of inflammatory cytokines and chemokines, such as IL-6 and IL-8, which recruit immune cells to the TME, further enhancing the inflammatory environment [90,91]. Interestingly, these altered KEGG pathways were upregulated in cells cultured under short-term hypoxia (1d) as well. Moreover, in areas of necrosis, necrotic cells trigger an inflammatory response [92,93] and promote the immune system [94].

A decreased expression of genes associated with metabolism, particularly lipid metabolic processes, was noted in prolonged hypoxic conditions, leading to significant alterations in cellular and systemic metabolic functions. These findings are somehow surprising given the fact that other studies show that cancer cells typically exhibit a metabolic reprogramming to adapt to the altered TME and sustain rapid proliferation by utilizing alternative energy sources such as anaerobic glycolysis (Warburg effect) [95], lipid metabolism [96], fatty acid oxidation [97], and/or glutamine metabolism [98]. Malignant cells modified their lipid metabolism to support rapid growth under inappropriate conditions like hypoxia and starvation. However, the precise mechanisms behind these metabolic alterations are currently not well understood.

A decreased expression of genes associated with lipid metabolism can indicate a remodeling of membrane lipids, which is a critical mechanism for metabolic adaptations in response to chronic hypoxia [99,100]. This process will reduce ATP consumption and production, which is essential for survival in low-oxygen conditions [100]. Furthermore, lipidomics and membrane biophysics identified changes in lipid composition and membrane properties under hypoxic conditions of pancreatic cancer cells, highlighting global lipidome alterations, namely a decrease in glycerophospholipids and sphingolipids, and an increase in cholesterol esters, triacylglycerides, and lipid storage under hypoxia, while the plasma membrane maintains its biophysical properties [101].

Moreover, hypoxia also induces alterations in the lipid metabolism of laryngeal carcinoma cells through the upregulation of the macrophage migration inhibitory factor (MIF)/IL-6/JAK-STAT pathway [102]. In the present study, although metabolism pathways in KEGG analysis are downregulated in prolonged exposure to hypoxia, the JAK-STAT signaling pathway remains upregulated, which may suggest that DH82 cells show evidence of adaptive responses via metabolic reprogramming.

This suggests that the effects of short-term and prolonged exposure to hypoxia differ, indicating a complex interplay between oxygen levels and cellular responses.

Another aspect considered in the present study was the effects due to different levels of nutrient deprivation, which vary across different tumor areas as well. The dysregulation observed in all DEGs during the period of short-term starvation (1d) were mainly related to GO terms in cellular responses to stimuli as well as signaling pathways. In contrast, prolonged starvation (3d) revealed a more pronounced alteration in gene expression patterns compared to controls, indicating that cellular adaptation mechanisms to starvation become prominent on day 3, simulating a central tumor zone.

Interestingly, our results show that prolonged exposure to starvation led to the overexpression of genes not only connected to angiogenesis (circulatory system development term), but also to metastasis (biological/cell adhesion terms). Moreover, the upregulation of the ERK1 and ERK2 cascade and its regulation were observed. The activation of ERK and AKT signaling pathways has also been found in canine histiocytic sarcoma cell lines through whole exome and transcription analysis in previous studies [51] and is linked to several cellular processes, such as cell cycle, proliferation, transcription, differentiation, survival, migration, adhesion, and metabolism [103]. In contrast to this study, however, a downregulation of terms associated with cell cycle and cell division and the cell cycle KEGG pathway was noted, suggesting that while cells decrease cell division, they may still engage in other processes that promote survival and migration, indicating a complex interplay between these pathways in the context of tumor progression.

One unexpected result emerged from the KEGG pathway enrichment analysis, indicating that the pathways in cancer (KEGG cfa05200) were affected by both, exposure to hypoxia and starvation, which was in the opposite direction. It was upregulated in hypoxia groups, while it was downregulated in the starvation condition, independent of the length of exposure, highlighting the distinct adaptations that cells undergo in response to varying environmental stresses.

Another notable finding was the significant overlap in upregulated DEGs between hypoxic and starving conditions, particularly in terms related to circulatory system development and cell adhesion. This suggests that both stressors may activate similar pathways that might contribute to tumor aggressiveness. The seven common genes identified in both conditions were *CCN2*, *FLRT3*, *CXCL10*, *FGFR2*, *SELE*, *CDH1*, and *CEACAM20*. Among the shared DEGs, several play an important role in numerous biological activities, which are crucial for tumor progression [104,105,106,107,108,109]. The C-X-C Motif Chemokine Ligand 10 (*CXCL10*) gene encodes for a pro-inflammatory cytokine involved in the immune system, apoptosis, and modulation of angiogenesis [104,105,106]. It has been linked to tumor progression in human pancreatic adenocarcinoma [108]. The E-selectin gene (*SELE*) is part of the selectin family of cell adhesion molecules, which is crucial for the adhesion of circulating tumor cells to the vascular endothelium, facilitating metastasis. *SELE* represents a prognostic factor for human colorectal cancer, with a high expression linked to a worsening of the prognosis [107]. In the present study, an increase in the expression of *CXCL10* under both hypoxia and starvation indicates its potential role in promoting tumor development in canine histiocytic sarcoma. Furthermore, the upregulation of *SELE* may synergistically enhance the metastatic potential of canine histiocytic sarcoma, highlighting their roles in tumor progression. These findings might indicate a similar increase in the biological aggressiveness of stressed neoplastic cells within central zones of solid tumors. Although information about the Carcinoembryonic Antigen-Related Cell Adhesion Molecule 20 (*CEACAM20*) gene is limited, CEACAM proteins are involved in cell adhesion, which links them to the metastatic process, and angiogenesis [109]. Another gene of potential interest is E-cadherin (*CDH1*), a transmembrane glycoprotein regulating cell-to-cell adhesion, which has been linked to tumor progression and metastasis in cases of decreased expression [110,111]. However, an unexpected finding in the present study was the upregulated expression of *CDH1*. It was upregulated under both hypoxia and starvation, suggesting a potential increase in cell adhesion that may influence tumor behavior by enhancing cell adhesion properties, potentially affecting metastatic processes. These findings suggest that the interplay between the E-selectin expression and the tumor microenvironment under hypoxia and starvation may influence the metastatic potential and overall prognosis of canine histiocytic sarcoma. The cellular communication network factor 2 (*CCN2*) is recognized for its role in numerous biological activities, including ECM remodeling and angiogenesis. *CCN2* expression increased in hypoxic pancreatic tumor cells to protect cells from hypoxia-mediated apoptosis [112]. Additionally, *CCN2* promotes the epithelial–mesenchymal transition (EMT), angiogenesis, and stroma infiltration in colorectal cancer [113] and enhances proliferation, migration, and invasion in urothelial bladder cancer [114]. Notably, *CCN2* is considered a promising therapeutic target, and several *CCN2* inhibitors, such as pamrevlumab (FG-3019), have already entered clinical trials for fibrotic disease and cancer [115,116,117]. The fibronectin leucine-rich transmembrane protein 3 (*FLRT3*) is involved in the regulation of the EMT [118]. Its expression is modulated by cancer-associated fibroblasts (CAFs) through the TGF-β/SMAD4 signaling pathway, which promotes aggressive cancer phenotypes [118]. The fibroblast growth factor receptor 2 (*FGFR2*) increases tumor cell proliferation and survival, and its overexpression is linked to aggressive tumors [119].

Based on these transcriptomic results, suppression of *CCN2*, *FGFR2*, and *SELE* may inhibit tumor proliferation, metastasis, or angiogenesis, while upregulation of *CDH1* and *CXCL10* could enhance cellular adhesion and antitumor immune responses, offering promising avenues for therapeutic intervention. Despite these insights, the study faces several limitations. The present investigation highlights changes in gene expression patterns. However, it will be crucial to further analyze these findings at the protein level in future studies. Therefore, no final conclusions about the functional in vivo relevance of these observations could be drawn. Thus, to validate the potential of these gene expression changes as therapeutic targets, further studies are needed that integrate in vitro functional assays, proteomics, and/or pharmacological inhibition to confirm the transcriptomic findings and assess effects on cell proliferation, migration, and survival. In addition, in vivo experiments would further support the translational relevance of these targets and evaluate their therapeutic feasibility and clinical significance. To summarize, hypoxia and starvation of canine HS cells influence various mechanisms, prominently involving genes associated with angiogenesis, metastasis, metabolism, as well as inflammation and immune responses in the central tumor zone, with angiogenesis and metastasis also occurring in the intermediate zone. While some of these responses, such as metabolic reprogramming and angiogenesis, may represent tumor-supportive adaptations, others—like the upregulation of immune-related genes—could reflect increased immunogenicity and enhanced tumor recognition by the host immune system. Additionally, a delayed response is observed in starvation. This delayed reaction may result from cells initially relying on residual intracellular metabolic resources before true starvation occurs. These findings highlight key transcriptomic changes that offer insights into both tumor adaptations and host-mediated defense mechanisms, which could serve as basic information for future functional insights.

## 4. Materials and Methods

### 4.1. Cell Culture

The present study investigated canine histiocytic sarcoma cells (DH82 cells), which were obtained from the European Collection of Authenticated Cell Cultures (ECACC No. 94062922). Cells were seeded in T25 flasks and cultured at a density of 0.33 × 10^6^ cells/flask in complete culture medium, consisting of minimal essential medium (MEM) with Earle’s salts (PAA, Cölbe, Germany) supplemented with 10% fetal calf serum (PAA), 1% penicillin/streptomycin (PAA), and 1% non-essential amino acids (Sigma-Aldrich, Taufkirchen, Germany). Cells were incubated in standard conditions (37 °C with 5% CO_2_, 21% O_2_ in a water-saturated atmosphere) as previously described [111]. After three adaptation days, cells were cultured under standard, hypoxic, and starving conditions for one day (short-term) and three days (prolonged exposure). For hypoxic conditions, flasks were transferred to a hypoxia chamber with 1% O_2_, ensuring stable low oxygen levels throughout the culture period. For starvation, the culture medium was replaced by a new medium as described above, but lacking fetal calf serum.

### 4.2. Proliferation Assay

For determination of cellular proliferation, the total number of cells was manually determined in a hemocytometer using trypan blue as previously described [120] at 1d and 3d under the different conditions and compared to the numbers of cells seeded. Per condition (control, hypoxia, starvation) six flasks were evaluated (*n* = 6 per time point and condition).

### 4.3. RNA Isolation

Total RNA was extracted and DNase digested and purified from DH82 cells cultured under the above-mentioned conditions as described before [121]. Briefly, cells were lysed in TRIzol^®^ (Invitrogen, Thermo Fisher Scientific, Langenselbold, Germany) and RNA was extracted using chloroform followed by a precipitation with isopropyl alcohol. The obtained RNA was cleaned up using the RNeasy Mini Kit (Qiagen, Hilden, Germany) in combination with on-column DNase digestion (RNAse free DNase Kit, Qiagen) according to the manufacturer’s instructions. The quality and integrity of total RNA was controlled using an Agilent Technologies 2100 Bioanalyzer (Agilent Technologies, Waldbronn, Germany). Three replicates per culture condition and time point were analyzed with RNA sequencing.

### 4.4. RNA Sequencing

A total of 100 ng of total RNA was used for the preparation of the RNA Sequencing library with NEBNext^®^ Single Cell/Low Input RNA Library Prep Kit for Illumina^®^ (New England Biolabs, Ipswich, MA, USA). The libraries were sequenced on Illumina NovaSeq 6000 (Illumina, Inc., San Diego, CA, USA) using the NovaSeq 6000 S1 Reagent Kit (Illumina, Inc., San Diego, CA, USA) (300 cycles, paired-end run 2 × 150 bp) with an average of 6 × 10^7^ reads per RNA sample.

### 4.5. Data Processing and Data Analysis

Reads from RNAseq were quality checked with FastQC (version 0.11.9, http://www.bioinformatics.babraham.ac.uk/projects/fastqc, accessed on 28 May 2024) [119], then trimmed using Trimgalore (version 0.6.7, https://www.bioinformatics.babraham.ac.uk/projects/trim_galore/, accessed on 28 May 2024) [122] with default settings. Trimmed reads were mapped to the dog genome (ENSMBL canis_lupus_familiaris, release 112) using the STAR aligner (version 2.5.2b, [123]) with default settings. Mapped reads were counted at the gene level using RsubRead (version 1.32.4, [124]).

The counts obtained from the dog genome mapping were normalized and log_2_-transformed by using iDEP2.01 (http://bioinformatics.sdstate.edu/idep/, accessed on 19 June 2024). The normalized data were then used for differentially expressed gene (DEG) analysis, hierarchical clustering analysis, and visualization by using iDEP2.01 (http://bioinformatics.sdstate.edu/idep/, accessed on 19 June 2024) [125,126]. Analysis was performed by using DESeq2. Genes with no gene symbols were removed, and genes showing an absolute log_2_ fold change > 1 and an adjusted *p*-value (Benjamini-Hochberg FDR) < 0.05 were selected as DEGs for this study. For identifying the common DEGs between each condition, the InteractiVenn (https://www.interactivenn.net/, accessed on 30 June 2024) online tool was used.

Principal component analysis (PCA) was performed on normalized expression data via DESeq2 to visualize sample clustering and the variance in gene expression across time and conditions.

### 4.6. Functional Enrichment Analysis

Enrichment analysis for gene ontology (GO) biological process terms, utilizing the GO FAT database, and Kyoto Encyclopedia of Genes and Genomes (KEGG) pathways were performed online using the Database for Annotation, Visualization, and Integrated Discovery (DAVID) (version Dec. 2021, Knowledgebase 195 v2023q4) (https://davidbioinformatics.nih.gov/, accessed on 20 September 2024) [127,128,129]. The GO FAT database contains more specific terms than the GO database [128]. Terms and pathways with a false discovery rate (FDR) of less than 0.05 were considered statistically significant. In this investigation, the overall strategies used were shown in Figure S1. 

### 4.7. Statistical Analysis

To ensure the reliability and rigor of statistical significance in the proliferation assay, all experiments were conducted in six biological replicates. Proliferation rates from day 0 to day 3 were expressed as median (min-max). The Friedman test was applied for global comparison of these three conditions (control, hypoxia, and starvation) to identify differences between time frames (day 0, 1, and 3) of each condition, and the Kruskal–Wallis test was used for overall comparisons to identify differences between conditions at the same time. Subsequently, the Wilcoxon Signed-Rank test and the Mann–Whitney U test with Bonferroni’s correction of the *p*-value were performed for pairwise comparisons of related and independent samples, respectively, to control for type I errors from multiple testing. *p*-values of less than 0.05 were considered statistically significantly different. All comparisons were performed using two-tailed tests. Statistical analysis was carried out using SPSS version 29 (SPSS, Inc, Chicago, IL, USA).

## Figures and Tables

**Table 1:** Cell proliferation assay results.

Conditions	Total Cell Number (×10^6^ cells/T25)			
	Day 1		Day 3	
	Median	Range	Median	Range
Control	6.58	5.50–6.99	6.58	5.75–7.85
Hypoxia	4.28	3.40–5.25	4.28	0.65–1.25
Starvation	5.85	4.65–8.05	5.85	6.00–7.75

**Table 2:** Number of differentially expressed genes in DH82 cells cultured under hypoxic, starving, and control conditions for different time frames.

Conditions	Pairwise Comparisons	Number of DEGs		
		Upregulated	Downregulated	Total
Short-term hypoxia (1d)	K_d1 versus H_d1	589	1056	1645
Short-term starvation (1d)	K_d1 versus S_d1	107	50	157
Prolonged hypoxia (3d)	K_d3 versus H_d3	681	620	1301
Prolonged starvation (3d)	K_d3 versus S_d3	249	587	836
Control over time	K_d1 versus K_d3	619	704	1323
Hypoxia over time	H_d1 versus H_d3	1606	1003	2609
Starvation over time	S_d1 versus S_d3	891	1258	2149

DEG: differentially expressed gene, DH82: canine histiocytic sarcoma cells, K: control, H: hypoxia, S: starvation, d1: day 1, d3: day 3.

**Table 3:** Differentially expressed genes and functional annotation clusters (GO terms) in DH82 cells cultured under various conditions compared to controls, ranked by false discovery rate.

Comparisons	Number of DEGs	Functional Annotation Clusters: GO Terms (FDR Value)
Short-term hypoxia (1d) (total: 1645)	Up: 589	Circulatory system development (<0.001), regulation of cell communication (<0.001), blood vessel morphogenesis (<0.001), vasculature development (<0.001), regulation of signal transduction (<0.001), cell adhesion (<0.001), angiogenesis (<0.001), regulation of cell migration (<0.001), regulation of cell motility (<0.001), positive regulation of angiogenesis (<0.001)
	Down: 1056	Cell cycle process (<0.001), cell cycle (<0.001), mitotic cell cycle process (<0.001), nuclear division (<0.001), chromosome segregation (<0.001), DNA metabolic process (<0.001), nuclear chromosome segregation (<0.001), DNA replication (<0.001), cell division (<0.001), DNA repair (<0.001)
Short-term starvation (1d) (total: 157)	Up: 107	Cellular response to chemical stimulus (<0.001), cell surface receptor signaling pathway (<0.05), negative regulation of response to stimulus (<0.05), second messenger-mediated signaling (<0.05), calcium-mediated signaling (<0.05)
	Down: 50	Cellular response to fibroblast growth factor stimulus (<0.05), response to fibroblast growth factor (<0.05)
Prolonged hypoxia (3d) (total: 1301)	Up: 681	Regulation of signal transduction (<0.001), cell surface receptor signaling pathway (<0.001), regulation of cell communication (<0.001), immune response (<0.001), positive regulation of response to stimulus (<0.001), cell adhesion (<0.001), biological adhesion (<0.001), inflammatory response (<0.001), vasculature development (<0.001), circulatory system development (<0.001)
	Down: 620	Lipid metabolic process (<0.001), cellular lipid metabolic process (<0.001), cell activation (<0.01), lipid biosynthetic process (<0.01), response to external stimulus (<0.01), cellular response to chemical stimulus (<0.01), fatty acid metabolic process (<0.01), regulation of cell communication (<0.05), regulation of lipid metabolic process (<0.05), membrane lipid metabolic process (<0.05)
Prolonged starvation (3d) (total: 836)	Up: 249	Cell surface receptor signaling pathway (<0.05), cell adhesion (<0.05), biological adhesion (<0.05), ERK1 and ERK2 cascade (<0.05), regulation of ERK1 and ERK2 cascade (<0.05), circulatory system development (<0.05), positive regulation of response to stimulus (<0.05), response to external stimulus (<0.05)
	Down: 587	Mitotic cell cycle (<0.001), mitotic cell cycle process (<0.001), cell cycle (<0.001), cell cycle process (<0.001), nuclear division (<0.001), chromosome segregation (<0.001), mitotic nuclear division (<0.001), mitotic sister chromatid segregation (<0.001), sister chromatid segregation (<0.001), cell division (<0.001)
Control, d1 vs. d3 (total: 1323)	Up: 619	n.s.
	Down: 704	Cell cycle process (<0.001), cell cycle (<0.001), mitotic cell cycle (<0.001), mitotic cell cycle process (<0.001), nuclear division (<0.001), mitotic nuclear division (<0.001), chromosome segregation (<0.001), chromosome organization (<0.001), DNA replication (<0.001), DNA metabolic process (<0.001)
Hypoxia, d1 vs. d3 (total: 2609)	Up: 1606	Regulation of gene expression (<0.001), transcription, DNA-templated (<0.001), regulation of RNA biosynthetic process (<0.001), RNA metabolic process (<0.001), RNA biosynthetic process (<0.001), regulation of response to stress (<0.001), positive regulation of metabolic process (<0.001), regulation of signal transduction (<0.001), regulation of apoptotic process (<0.001), regulation of defense response (<0.001)
	Down: 1003	Cell adhesion (<0.001), cell migration (<0.001), cell motility (<0.001), regulation of immune system process (<0.001), endocytosis (<0.001), cell activation (<0.001), response to external stimulus (<0.001), vasculature development (<0.001), angiogenesis (<0.001), phagocytosis (<0.001)
Starvation, d1 vs. d3 (total: 2149)	Up: 891	Lipid metabolic process (<0.001), single-organism catabolic process (<0.001), ion transport (<0.05), cell–cell signaling (<0.05), movement of cell or subcellular component (<0.05), transmembrane transport (<0.05), regulation of phosphate metabolic process (<0.05), regulation of protein phosphorylation (<0.05), regulation of cell communication (<0.05), regulation of cellular component movement (<0.05)
	Down: 1258	Cell cycle (<0.001), cell cycle process (<0.001), mitotic cell cycle (<0.001), nuclear division (<0.001), chromosome segregation (<0.001), chromosome organization (<0.001), DNA metabolic process (<0.001), DNA replication (<0.001), DNA repair (<0.001), cell migration (<0.001)

DH82: canine histiocytic sarcoma cells, FDR: false discovery rate, n.s.: no significantly enriched gene ontology (GO) terms.

**Table 4:** Differentially expressed genes and functional annotation clusters (KEGG pathways) in DH82 cells cultured under various conditions, compared to controls, ranked by false discovery rate.

Comparisons	Number of DEGs	Functional Annotation Clusters: KEGG Pathways (FDR Value)
Short-term hypoxia (1d) (total: 1645)	Up: 589	AGE-RAGE signaling pathway in diabetic complications (<0.001), Pathways in cancer (<0.001), HIF-1 signaling pathway (<0.001), TNF signaling pathway (<0.01), MAPK signaling pathway (<0.01), NF-κB signaling pathway (<0.01), Focal adhesion (<0.01), PI3K-Akt signaling pathway (<0.05), Endocytosis (<0.05), MicroRNAs in cancer (<0.05)
	Down: 1056	Cell cycle (<0.001), Fanconi anemia pathway (<0.001), DNA replication (<0.001), Homologous recombination (<0.01), Mismatch repair (<0.01)
Short-term starvation (1d) (total: 157)	Up: 107	n.s.
	Down: 50	Complement and coagulation cascades (<0.001), Pathways in cancer (<0.05)
Prolonged hypoxia (3d) (total: 1301)	Up: 681	IL-17 signaling pathway (<0.001), TNF signaling pathway (<0.001), Cytokine–cytokine receptor interaction (<0.001), Pathways in cancer (<0.001), NF-κB signaling pathway (<0.001), C-type lectin receptor signaling pathway (<0.001), Cell adhesion molecules (<0.001), MAPK signaling pathway (<0.001), JAK-STAT signaling pathway (<0.001), NOD-like receptor signaling pathway (<0.001)
	Down: 620	Metabolic pathways (<0.001)
Prolonged starvation (3d) (total: 836)	Up: 249	Complement and coagulation cascades (<0.05)
	Down: 587	Cell cycle (<0.001), Pathways in cancer (<0.05)
Control, d1 vs. d3 (total: 1323)	Up: 619	n.s.
	Down: 704	Cell cycle (<0.001), DNA replication (<0.001), Homologous recombination (<0.001), Mismatch repair (<0.001), Steroid biosynthesis (<0.01), Base excision repair (<0.01), Pyrimidine metabolism (<0.01), p53 signaling pathway (<0.05), Transcriptional misregulation in cancer (<0.05), IL-17 signaling pathway (<0.05)
Hypoxia, d1 vs. d3 (total: 2609)	Up: 1606	Cytokine–cytokine receptor interaction (<0.01), JAK-STAT signaling pathway (<0.05), Neurotrophin signaling pathway (<0.05), TNF signaling pathway (<0.05)
	Down: 1003	Focal adhesion (<0.001), ECM–receptor interaction (<0.001), Steroid biosynthesis (<0.001), Proteoglycans in cancer (<0.01), Phagosome (<0.01), Rap1 signaling pathway (<0.01), Endocytosis (<0.01), PI3K-Akt signaling pathway (<0.01), Metabolic pathways (<0.05), Glutathione metabolism (<0.05)
Starvation, d1 vs. d3 (total: 2149)	Up: 891	n.s.
	Down: 1258	Cell cycle (<0.001), DNA replication (<0.001), ECM–receptor interaction (<0.01), Cytokine–cytokine receptor interaction (<0.01), PI3K-Akt signaling pathway (<0.05), Motor proteins (<0.05), Pyrimidine metabolism (<0.05), Mismatch repair (<0.05), Base excision repair (<0.05), Steroid biosynthesis (<0.05)

DH82: canine histiocytic sarcoma cells, FDR: false discovery rate, n.s.: no significantly enriched KEGG pathways.

## Data Availability

Raw sequence reads and a normalized expression matrix are available at the GEO public database (GEO) [130], ID: GSE183324.

## Supplementary Materials

The following supporting information can be downloaded at https://www.mdpi.com/article/10.3390/ijms26146629/s1.

## Abbreviations

The following abbreviations are used in this manuscript:

CNS	Central nervous system
DEGs	Differentially expressed genes
DH82	Canine histiocytic sarcoma cells
ECM	Extracellular matrix
EMT	Epithelial-to-mesenchymal transition
FDR	False discovery rate
GO	Gene ontology
H	Hypoxia
HIF	Hypoxia inducible factor
HS	Histiocytic sarcoma
IL	Interleukin
K	Control
KEGG	Kyoto Encyclopedia of Genes and Genomes
MRI	Magnetic resonance imaging
NF-κB	Nuclear factor kappa
PC	Principal component
PET	Positron emission tomography
pO_2_	Oxygen partial pressure
S	Starvation
SPECT	Single-photon emission computed tomography
TME	Tumor microenvironment
TNF	Tumor necrosis factor
