# Supplementary figures and images for: Transcriptomic Alterations of Canine Histiocytic Sarcoma Cells in Response to Different Stressors
Source: Int J Mol Sci. 2025 Jul 10;26(14):6629. doi: 10.3390/ijms26146629 (PMC12294338; doi:10.3390/ijms26146629)

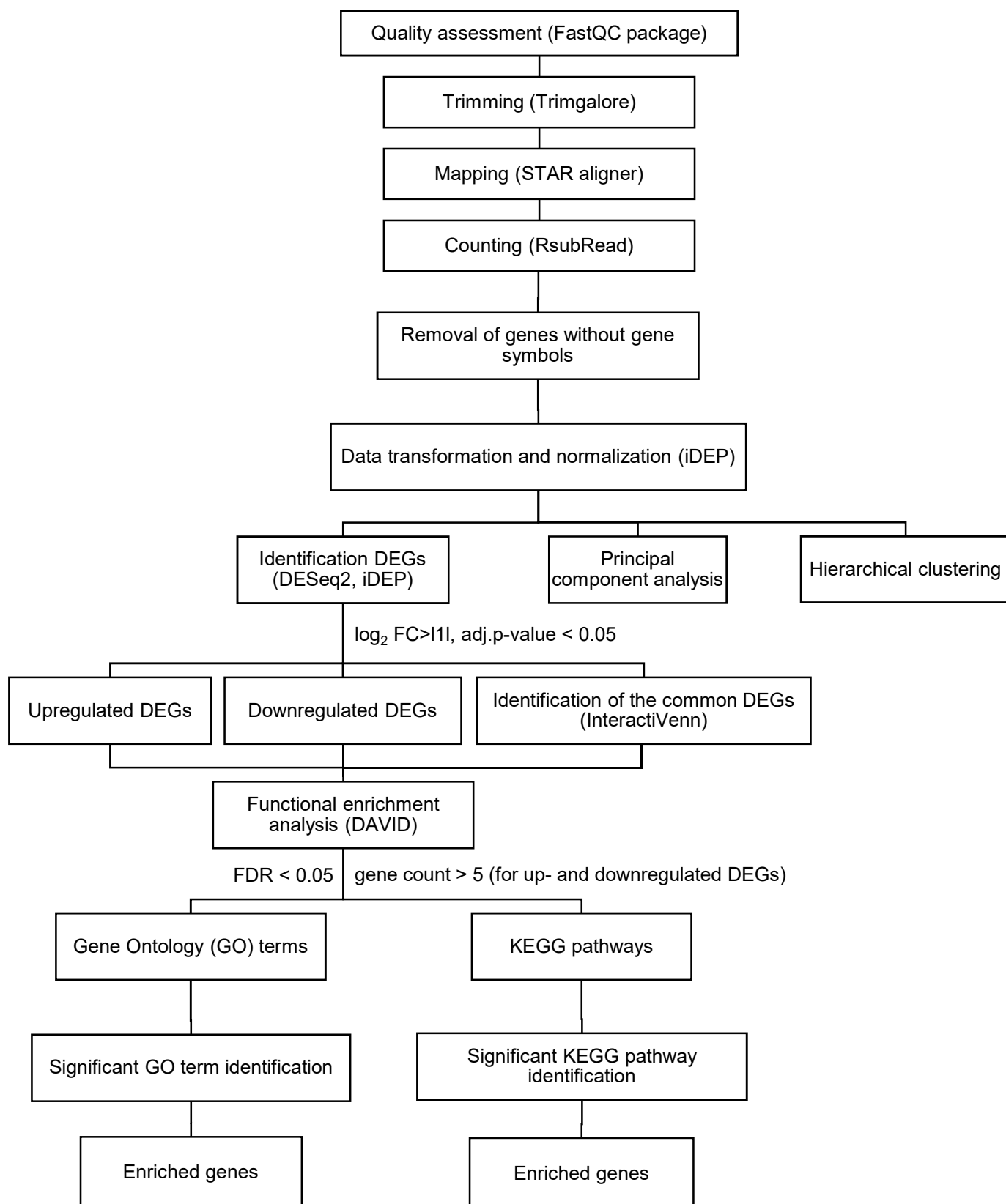

**Figure S1.** Workflow diagram of strategies used in this study

Supplement: Supplementary file 1 [file ijms-26-06629-s001.zip › Figure S1_Workflow diagram of strategies used in this study.pdf]

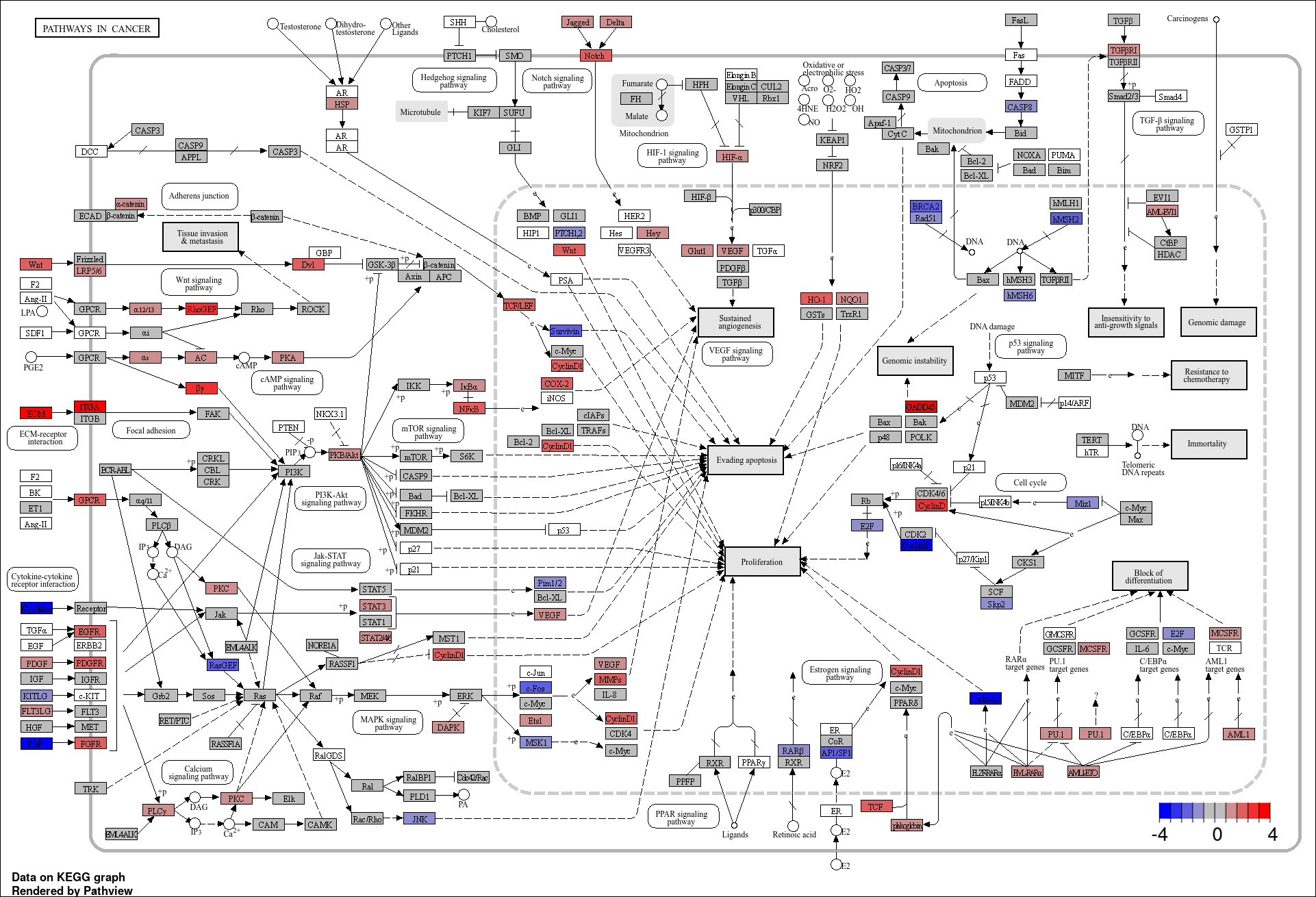

Supplement: Supplementary file 1 [file ijms-26-06629-s001.zip › Figure S2. Display of pathways commonly involved in cancer-short-term hypoxia.jpg]

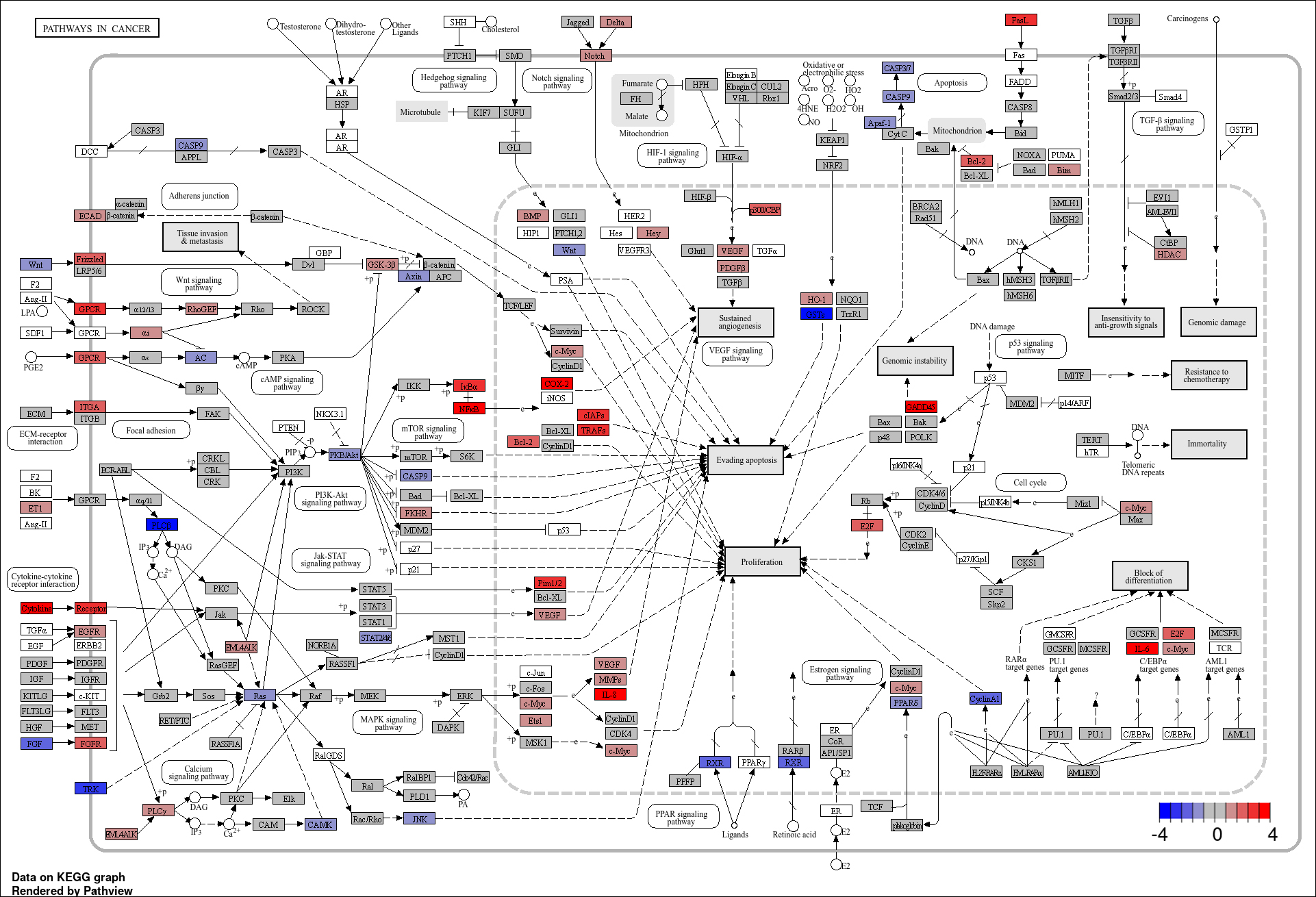

Supplement: Supplementary file 1 [file ijms-26-06629-s001.zip › Figure S3. Display of pathways commonly involved in cancer-prolonged hypoxia.jpg]

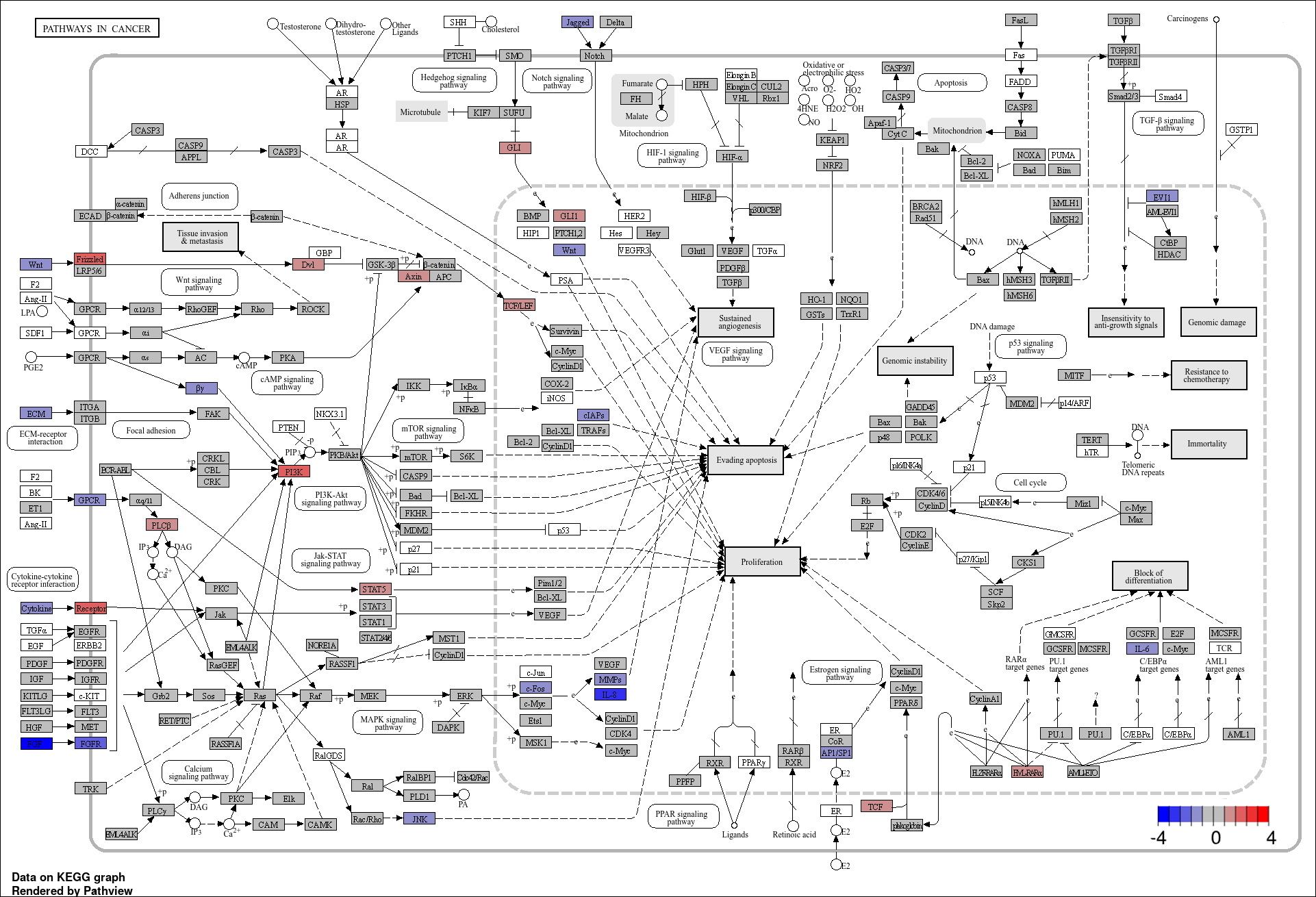

Supplement: Supplementary file 1 [file ijms-26-06629-s001.zip › Figure S4. Display of pathways commonly involved in cancer-short-term starvation.jpg]

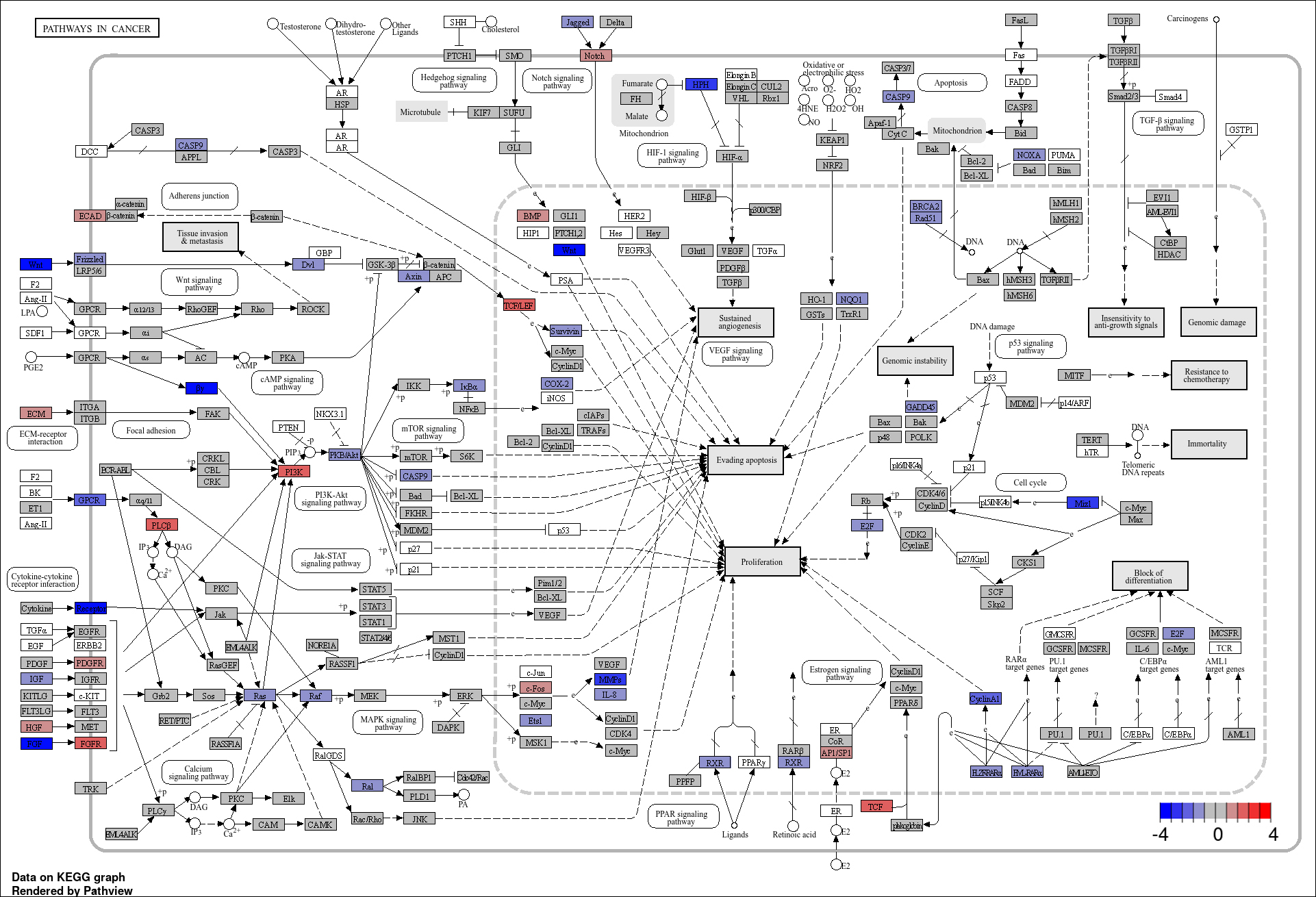

Supplement: Supplementary file 1 [file ijms-26-06629-s001.zip › Figure S5. Display of pathways commonly involved in cancer-prolonged starvation.jpg]
